# Plasma C4d Correlates With C4d Deposition in Kidneys and With Treatment Response in Lupus Nephritis Patients

**DOI:** 10.3389/fimmu.2020.582737

**Published:** 2020-10-02

**Authors:** Myriam Martin, Rebecca Trattner, Sara C. Nilsson, Albin Björk, Agneta Zickert, Anna M. Blom, Iva Gunnarsson

**Affiliations:** ^1^Department of Translational Medicine, Section of Medical Protein Chemistry, Lund University, Malmö, Sweden; ^2^Region Skåne, Laboratory Medicine, Clinical Chemistry, Malmö, Sweden; ^3^Department of Medicine, Division of Rheumatology, Karolinska Institutet, Stockholm, Sweden; ^4^Rheumatology, Karolinska University Hospital, Stockholm, Sweden

**Keywords:** systemic lupus erythematosus, complement, C4d, lupus nephritis, treatment response, kidney deposition

## Abstract

**Objective:**

To examine whether C4d plasma levels correlate with treatment response and C4d kidney deposition in systemic lupus erythematosus (SLE) with lupus nephritis (LN).

**Methods:**

C4d plasma levels were analyzed by a unique assay specifically detecting C4d arising from complement activation and C4 plasma levels were quantified with competitive ELISA. SLE patients with LN (71) and active SLE patients without LN (22) plus 145 controls were included. For 52 LN patients samples were available both at baseline and after immunosuppressive treatment. C4d kidney deposition was detected using immunohistochemistry in two matching kidney biopsies of 12 LN patients.

**Results:**

In comparison to population-based controls, plasma C4d levels were significantly increased in SLE patients (0.33 mg/L versus 0.94 mg/ml, *p* < 0.0001) with significantly higher levels in LN patients (1.02 mg/L) than in non-renal SLE patients (0.57 mg/L, *p* = 0.004). The C4d/C4 ratio was also significantly higher in LN (11.2) than in non-renal SLE patients (2.5, *p* = 0.0002). According to ROC curve analysis, C4d was found to be an accurate marker to discriminate LN from non-renal SLE patients (*p* = 0.004). The C4d/C4 ratio displayed even higher specificity, sensitivity and overall accuracy as marker for LN than C4d and C4 alone. At baseline, C4d levels correlated significantly with urine-albumin to creatinine ratio (*r*_*s*_ = 0.43, *p* = 0.011) and with renal activity index (*r*_*s*_ = 0.37, *p* = 0.002). Immunohistochemical staining showed glomerular deposits of C4d in kidney biopsies, which strikingly correlated with plasma C4d levels (*r*_*s*_ = 0.7, *p* = 0.0002). Plasma C4d declined significantly after treatment in patients that experienced favorable clinical and histopathological response (*p* < 0.0001), while levels remained mainly unchanged in non-responders.

**Conclusion:**

Plasma C4d discriminates LN from active non-renal SLE, correlates with C4d kidney deposits and appears valuable in monitoring responsiveness to various treatments. The C4d/C4 ratio might be superior to C4d alone.

## Introduction

Systemic lupus erythematosus (SLE) is a chronic autoimmune disorder with heterogeneous manifestations. Despite advanced treatment strategies, lupus nephritis (LN) remains one of the most common organ-threatening manifestations with significant morbidity and mortality (1). Early diagnosis and initiation of treatment is of uttermost importance for the prognosis. Renal biopsy is the gold standard to diagnose and classify LN as well as to guide therapy (2–4). The overarching treatment goal is to prevent renal failure and optimally to achieve complete clinical remission and maintain it long-term (5). To monitor responsiveness to immunosuppressive treatment, different scoring systems mainly based on the reduction of proteinuria can be applied (2, 6).

Repeated renal biopsies have been suggested to improve the evaluation of treatment response in LN (7). The downside is that renal biopsies are invasive and bear the risk of complications. Thus, less invasive biomarkers for renal disease activity are desirable. We previously showed that C4d, which is the final cleavage fragment of C4 arising from complement activation, is a superior marker to C4 in identifying LN flares and that C4d, but not C3 and C4, can forecast recurrence of LN (8). Levels of C3 and C4 are the net result of complement activation and rate of synthesis, whereas C4d is exclusively generated upon complement activation, which strengthens its suitability as biomarker. Further, it has a favorable half-life compared to other complement activation products such as C3a or C5a. C4d measurement is robust, easy and highly specific with our assay (9).

In the current study, we confirmed the value of C4d as a biomarker for LN in an independent SLE cohort. We revealed that plasma C4d levels correlate with C4d deposited in kidney biopsies of LN patients and that plasma C4d levels associate with both histopathological and clinical treatment response at follow-up. Thus, the simple measurement of C4d plasma levels might be used to improve the evaluation of treatment outcome.

## Materials and Methods

### SLE Patients and Control Populations

In total, 93 patients with diagnosed SLE and classified according to the American College of Rheumatology classification criteria and/or the Systemic Lupus International Collaboration Clinics (SLICC) classification criteria were included in the study (10, 11). In 71 patients, samples were obtained at the time-point of renal biopsy. The renal biopsies were classified according to the International Society of Nephrology/Renal Pathology Society (ISN/RPS) classification (see Table 1) (12). Activity and chronicity index were evaluated in the renal biopsies (13). There were two patients with ISN/RPS class II (mesangial LN), 51 patients with a proliferative pattern (class III and IV+V) and 18 patients with a pure membranous LN (class V). In 52 of the patients with LN, repeated biopsies were collected after three to fourteen (median eight) months to evaluate the histopathological response.

**TABLE 1:** Classification of renal biopsies according to the International Society of Nephrology/Renal Pathology Society (ISN/RPS) classification.

ISN/RPS class	Baseline	Post-treatment
I-II	0	14
III (A)	9	0
III (A/C)	5	5
III (A)+V	5	1
III (A/C)+V	1	0
III (C)	0	5
IV-S (A)	4	1
IV-S (A/C)	3	0
IV-S (A/C)+V	1	0
IV-G (A)	7	1
IV-G (A/C)	4	1
IV-G (A)+V	2	1
IV-G (A/C)+V	0	1
V	11	19
V+II	0	3

*ISN/RPS, International Society of Nephrology/Renal Pathology Society.*

Twenty-two of the SLE patients had active SLE with no signs of renal involvement and were used as non-renal SLE controls. Disease activity in the non-renal SLE patients was estimated using the SLE Disease Activity Index 2000 (SLEDAI-2K) (14). The median score was 8 (range 2–18).

As disease controls, fourteen patients with biopsy-proven IgA nephropathy were included. Blood samples from 145 population-based controls were also included. Demographics of all participants are shown in Table 2.

**TABLE 2:** Demographics of patients and controls.

Characteristics	Controls *n* = 145	IgAN *n* = 14	LN (SLE) *n* = 71	Non-renal (SLE) *n* = 22
Age	41 (16–79)	48 (20–72)	33 (18–79)	46 (18–65)
Females, n (%)	114 (79%)	7 (50%)	62 (86%)	22 (100%)
C4d (mg/L)	0.33 (0–1.55)	0.30 (0.05–1.39)	1.02 (0.15–3.03)	0.57 (0–1.71)
C3 (g/L)	n. d.	n. d.	0.57 (0.12–1.38) 9 missing	0.82 (0.41–1.24)
C4 (mg/L)	n. d.	n. d.	86 (3.15–554)	231 (3.15–441)
C4d/C4 ratio	n. d.	n. d.	11.2 (0.47–930)	2.49 (0–527)
Creatinine (μmol/L)	n. d.	n. d.	75 (32–188)	64 (55–161)
Activity index	n. d.	n. d.	5 (0–12)	n.d.
Chronicity index	n. d.	n. d.	0 (0–7)	n.d.
Urine-albumin to creatinine ratio (mg/mmol)	n.d.	n.d.	124 (0–556) 37 missing	n.d.
Urine-albumin >0.5 g/day, n (%)	n.d.	n.d.	56 (79%)	n.d.
Anti-dsDNA-Ab positive, n (%)	n. d.	n. d.	49 (78%) 8 missing	17 (77%)
Prednisolone, n (%) (mg/day)	n.d.	8 (57.1%) 5 (0–30)	45 (63%) 7.5 (0–60)	11 (50%) 2.5 (0–30)
DMARD, n (%)	n. d.	Any 3 (21.4%), AZA 1, CYC 1, INF 1	Any 14 (20%), AZA 6, MMF 4, CYC 2, MTX 2	Any 9 (41%), AZA 3, MMF 2, CYC 3, MTX 1
Antimalarials, n (%)	n. d.	0	19 (27%)	12 (55%)

*Values are presented as median (range) unless otherwise stated. AZA, azathioprine; CYC, cyclophosphamide; DMARD, disease-modifying anti-rheumatic drugs; MMF, mycophenolate mofetil; MTX, methotrexate; IFN, infliximab; n.d., not determined.*

Collection of samples and healthy control subjects was performed according to the Declaration of Helsinki and approved by the Regional Ethical Review Boards in Stockholm (Dnr. 2012/1550, 2014/1337) with all participants providing written consent to participate in the study.

### Enzyme Immunoassay for Assessment of Complement C4d

Plasma C4d levels were measured with Complement C4d assay (#COMPL C4d RUO, SVAR Life Science) according to the manufacturer’s instruction. The normal reference range is defined as 0.03–0.15 mg/L. Absorbance was measured in Cytation-5 multi-mode reader (BioTek).

### Competitive Immunoassay for Assessment of Complement C4

Plasma C4 levels were determined with Human Complement C4 ELISA Kit (#ab108824, Abcam) according to the manufacturer’s instruction. The normal reference range is defined as 160–480 mg/L. Absorbance was measured as above. Values below the detection limit were set to 3.15 mg/L for statistical calculations.

### Immunohistochemical Staining of C4d in Kidney Biopsies

In order to test the specificity of the antibody, Daudi cells coated with C4b and C4d were prepared. The cells were incubated in DGVB^++^ (2.5 mM veronal buffer pH 7.3, 72 mM NaCl, 140 mM glucose, 0.1% gelatin, 1 mM MgCl_2_, and 0.15 mM CaCl_2_) with 1 μM recombinantly expressed OmCI (to prevent lysis of cells), 50 μg/ml ofatumumab recognizing CD20 expressed on cells (GlaxoSmithKline) and 5% normal human serum, 5% heat-inactivated human serum or 5% factor I depleted human serum (Complement Tech). The cells were then formalin-fixed and paraffin-embedded. Normal human serum was prepared as previously described (15) with approval of the Regional Ethical Review Boards in Lund (Dnr. 2013/846). Heat-inactivated was achieved by incubation at 56°C for 30 min. Paraffin-embedded kidney biopsies and controls were cut in 4 μm sections and pre-treated using the PT-link system (Dako) with antigen retrieval at pH 9 (Envision Flex high pH kit, Dako). C4d deposition was detected with the same anti-C4d-neo monoclonal Ab (dilution 1:5000, SVAR Life Science) as used in the C4d assay. Biopsies were stained for 30 min using Envision Flex (Dako) reagents in an Autostainer Plus system according to the manufacturer’s protocol (Dako). Intensity of the staining in glomeruli was scored from zero (negative) to three (high) by two researchers and one pathologist in a blinded manner. Kidney biopsies were only scored if at least two glomeruli were present. Kidney C4d scores for individual patients are presented in Table 3.

**TABLE 3:** Comparison of plasma C4d levels, kidney C4d score and LN classes.

	Baseline			Post-treatment		
Patient #	C4d (mg/L)	Kidney score	ISN/RPS class	C4d (mg/L)	Kidney score	ISN/RPS class
P1	0.686	2	V	1.208	3	V
P2	3.033	2	IV-S (A)	0.385	0	I
P3	1.154	2	IV-G (A)+V	0.551	3	V
P4	1.002	3	V	1.881	3	III (A)+V
P5	2.104	2	IV-G (A)	0.279	0	II
P6	2.928	3	IV-G (A)+V	0.746	3	IV-G (A)+V
P7	0.807	2	III (A)	0.225	0	II
P8	0.706	1	III (A)	0.315	1	II
P9	1.173	0	III (A/C)	0.293	0	V
P10	1.386	2	IV-G (A)	0.590	1	V
P11	0.789	1	IV-S (A/C)	0.272	1	II
P12	1.100	1	III (A)	0.354	0	II

*LN, lupus nephritis; ISN/RPS, International Society of Nephrology/Renal Pathology Society.*

### Standard Laboratory Tests and Reference Values

Plasma creatinine was analyzed according to clinical routine at the Karolinska University Hospital and expressed as micromole/liter. Renal function was estimated using the Modification of Diet in Renal Disease (MDRD) study equation for estimating glomerular filtration rate (16).

For analysis of albuminuria, either 24-h urinary collections (grams/24 h) or urine-albumin to creatinine ratios (u-ACR) (mg/mmol) were performed. Being a retrospective study, methods for estimation of albuminuria have changed over time and some data regarding u-ACR for the entire cohort is thereby missing. In order to analyze response to therapy, we have thus estimated the grade of proteinuria (<0.2 g/day or < 0.5 g/day) by the method available at the biopsy time-point.

Anti-dsDNA antibodies were analyzed according to CLIFT, ELISA or multiplex methods used as routine at the Department of Clinical Immunology at the Karolinska University Hospital, method depending on the analysis available at the inclusion time-point. As different methods were used, we chose to define anti-dsDNA as positive or negative.

### Definitions of Response to Treatment

We defined complete clinical response (CR) as having an inactive urinary sediment, proteinuria ≤0.2 g/day and normal glomerular filtration rate >90 mL/min or stable (within 10% of normal if previously abnormal) renal function. Partial response (PR) was defined by having an inactive sediment, proteinuria ≤0.5 g/day and normal or stable (<10% deterioration from baseline if previously abnormal) renal function. Patients not reaching the above criteria were regarded as clinical non-responders (NR) (2).

Using repeated renal biopsies for evaluation of response, we defined histopathological response (HR) as transformation into class I, II or III/IV-C whereas persistent class III/IV-A or III/IV-A/C and persistent, or transformation into, class V was considered as histopathological non-response (HNR) (17).

### Statistical Analyses

The control and patient data did not pass the normality test, thus nonparametric statistics were applied. Mann-Whitney U and Kruskal-Wallis rank-sum tests were used to calculate statistical significance of nonparametric continuous data, which are displayed as median with 25–75% quantiles plus whisker and listed as median (range). Wilcoxon matched-pairs signed rank test was applied for comparison of baseline and post-treatment measurements. Spearman’s rank-order correlation test was used to analyze correlations of nonparametric data. ROC curve analysis was used to categorize C4d and C4 levels. To evaluate accuracy, the AUC of ROC curves as well as sensitivity, specificity, positive predictive values (PPVs) and negative predictive values (NPVs) were determined. McNemar’s test was used for comparison of two markers in terms of accuracy. To estimate the relative risk, odds ratios (ORs) and 95% confidence intervals (CIs) were calculated by logistic regression. A statistical significance level (*p* value) < 0.05 was defined as statistically significant. Analyses were carried out using JMP Pro 12 (SAS Institute) and IBM SPSS Statistics 25 (IBM) software.

## Results

### Plasma C4d Levels Are Higher in Lupus Nephritis Patients Than in Non-renal SLE Patients

**FIGURE 1:**
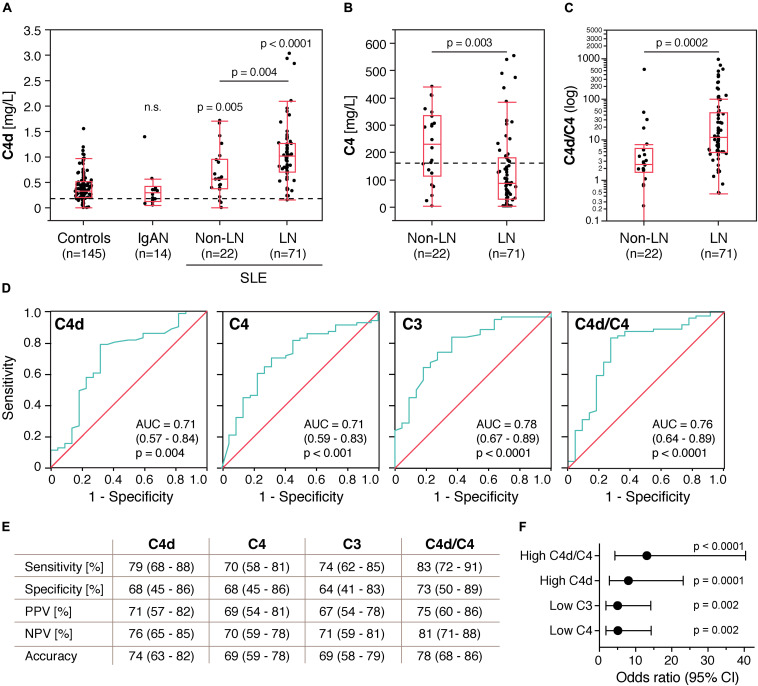
Plasma C4d levels are increased in patients with systemic lupus erythematosus (SLE) and discriminate lupus nephritis (LN) from non-renal SLE patients. **(A)** C4d levels in control subjects, IgA nephropathy patients (IgAN) and SLE patients without and with lupus nephritis. **(B,C)** C4 levels and C4d/C4 ratio in non-renal SLE patients and LN patients. Data are presented as medians with 25–75% quantiles plus whiskers, and significance was calculated using Kruskal-Wallis rank-sum and Mann–Whitney U tests. The C4d/C4 ratio for one of the non-renal SLE patients is zero and can thus not be displayed on a logarithmic scale. Dotted lines indicate the upper normal reference range for C4d (0.15 mg/L) and the lower normal reference range for C4 (160 mg/L). **(D)** Area under the ROC curve analysis showing accuracy of C4d, C3, C4, and C4d/C4 ratio as markers for LN. **(E)** Sensitivity, specificity, positive predictive value (PPV), negative predictive value (NPV) and overall accuracy for C4d, C3, C4, and C4d/C4 ratio as markers for LN. The disease prevalence was set to 50%. Data are presented with 95% CIs. **(F)** Association of high C4d (>0.67 mg/L), low C3 (<0.77 g/L), low C4 (<154 mg/L) and high C4d/C4 ratios (>4.19) with LN. Significance was calculated using binary logistics, and ORs are indicated with a dot connected to the 95% CI.

Plasma C4d levels were determined by a commercial complement C4d assay, which is a further development of our in-house ELISA (9). The median C4d level of population-based controls was 0.33 mg/L (0–1.55 mg/L) (Figure 1A). Fourteen IgA nephropathy patients were included as disease controls and showed C4d levels in the same range (0.30 mg/L, 0.05–1.39 mg/L). In comparison to the population-based controls, C4d levels were significantly increased in SLE patients (*p* < 0.0001), with significantly higher C4d levels in LN patients (1.02 mg/L, 0.15–3.03 mg/L) than in non-renal SLE patients (0.57 mg/L, 0–1.71 mg/L, *p* = 0.004). As expected, LN patients had significantly lower C4 levels (86 mg/L, 3.15–554 mg/L) than non-renal SLE patients (231 mg/L, 3.15–441 mg/L, *p* = 0.003) (Figure 1B). The C4d/C4 ratio was additionally calculated and displayed significant difference between LN (11.2, 0.47–930) and non-renal SLE patients (2.49, 0–527, *p* = 0.0002) (Figure 1C).

### C4d Levels Correlate With LN

ROC curve analysis showed that C4d likewise C3 and C4 exhibited statistically significant accuracy as markers for LN. The AUC of the C4d/C4 ratio was higher than for C4d or C4 alone, but slightly lower than for C3 (Figure 1D). C4d, C4 and the C4d/C4 ratio were categorized according to the ROC curve analyses. Routine clinical reference values were applied to categorize C3. C4d levels above 0.67 mg/L were defined as high. Applying this cut-off, 79% of LN patients, 32% of non-renal SLE patients and less than 18% of the controls had high C4d levels, confirming the validity of this cut-off. C4 levels below 154 mg/L were defined as low and C4d/C4 ratios above 4.19 were defined as high.

Validating the suitability as biomarker for LN further, we found that high C4d levels exhibited higher sensitivity, specificity, positive predictive value (PPV), negative predictive value (NPV) and overall accuracy than low C3 and C4 levels (Figure 1E). However, according to McNemar’s test, high C4d levels alone were not statistically superior to low C4 levels (*p* = 0.31), but high C4d/C4 ratios, which were even more sensitive and specific than high C4d levels alone, exhibited statistically superior accuracy as a marker for LN than low C4 (*p* = 0.012) and similar to high C4d (*p* = 0.58). McNemar’s test could not be performed for comparisons to low C3 levels, since C3 levels were not determined for nine LN patients. OR analysis revealed that high C4d (OR 8.0, *p* = 0.0001), low C3 (OR 5.03, *p* = 0.002) and low C4 (OR 5.1, *p* = 0.002) levels as well as high C4d/C4 ratios (OR 13.1, *p* < 0.0001) all associated significantly with LN and that high C4d/C4 ratios exhibited the highest relative odds to occur if the patient has LN (Figure 1F).

### Correlation of Plasma C4d Levels With Other Clinical Variables

**FIGURE 2:**
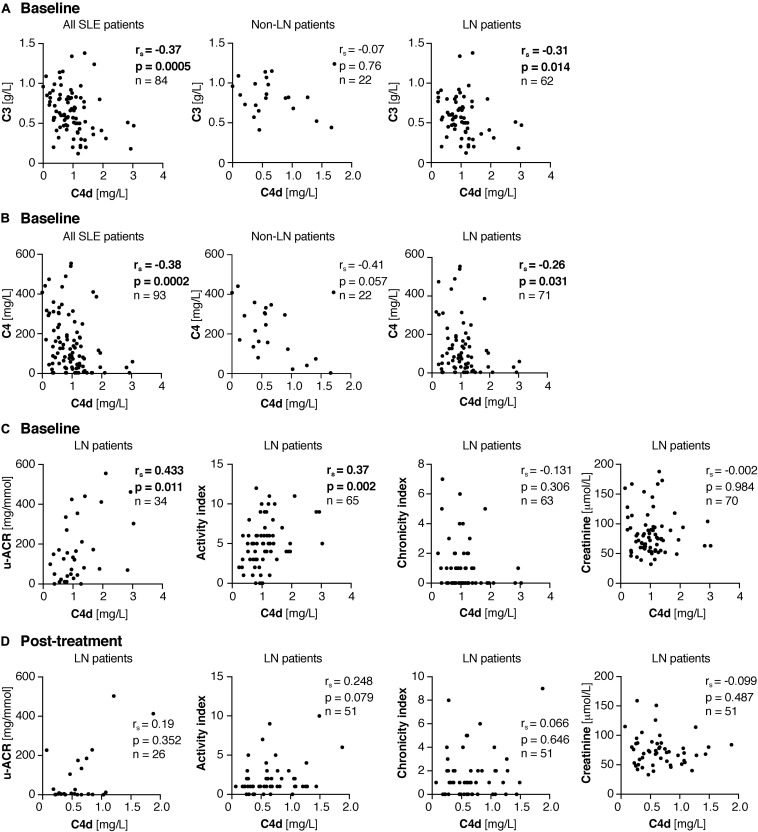
Correlations of plasma C4d levels with clinical variables. **(A,B)** Correlations of plasma C4d with C3 **(A)** and C4 **(B)** in all 93 SLE patients as well as split up in the 22 non-LN and 71 LN patients at baseline. **(C,D)** Correlation of plasma C4d with u-ACR, activity and chronicity index as well as creatinine at baseline **(C)** and post-treatment **(D)**. **(A–D)** Not all variables were determined for all patients; therefore n is shown in each correlation graph separately. Significant correlation coefficients and *p* values are bold. Significance was calculated using Spearman’s rank-order correlation test. u-ACR, urine-albumin to creatinine ratio.

At baseline, C4d correlated negatively with C3 (*r*_*s*_ = –0.37, *p* = 0.0005, values for nine LN patients were not determined) and C4 (*r*_*s*_ = –0.38, *p* = 0.0002) in all 93 SLE patients (Figures 2A,B). Interestingly, C4d did not correlate significantly with C3 (*r*_*s*_ = –0.07, *p* = 0.76) and C4 (*r*_*s*_ = –0.41, *p* = 0.057) in the 22 non-renal SLE patients, but only in the 71 LN patients [C3 (*r*_*s*_ = –0.31, *p* = 0.014), C4 (*r*_*s*_ = –0.26, *p* = 0.031)] (Figures 2A,B).

Data regarding urine-albumin to creatinine ratio (u-ACR) was available in 34 of the LN patients, either at baseline in patients with repeated biopsies (*n* = 25) or among LN patients with single renal biopsy samples only (*n* = 9). In these, C4d correlated strongly with u-ACR (*r*_*s*_ = 0.433, *p* = 0.011). Additionally, C4d correlated significantly with the renal activity index (*r*_*s*_ = 0.37, *p* = 0.002), but not with chronicity index or creatinine level (Figure 2C).

In all SLE patients as well as in the LN patients at baseline, neither prednisolone, antimalarial or disease-modifying anti-rheumatic drugs influenced C4d levels. However, in the non-renal SLE patients, patients on disease-modifying anti-rheumatic drugs had significantly lower C4d levels than patients not receiving this medication (0.39 mg/L versus 0.66 mg/L, *p* = 0.042). C4d levels were slightly, but insignificantly higher in LN patients with occurrence of anti-dsDNA-Abs than in LN patients without (1.05 mg/L versus 0.86 mg/L, *p* = 0.175).

Post-treatment, data on u-ACR was available in 26 of the 52 patients. In these, C4d levels did not correlate with the u-ACR. Furthermore, there was no association with activity and chronicity index, or with the creatinine levels (Figure 2D).

Correlations of the C4d/C4 ratio with clinical variables of the 52 re-biopsied LN patients at baseline and post-treatment are listed in Table 4.

**TABLE 4:** Correlation of plasma C4d/C4 ratio with clinical variables at first and second biopsy of the 52 re-biopsied LN patients.

C4d/C4 ratio correlation with	r_*s*_	*P* Value	n
**Baseline**			
C3	−0.475	**0.0007**	47
u-ACR	0.415	**0.0389**	25
Activity index	0.261	0.0674	50
Chronicity index	−0.330	**0.0192**	50
Creatinine	0.044	0.7595	51
**Post-treatment**			
C3	−0.532	**0.0002**	44
u-ACR	0.062	0.7630	26
Activity index	0.191	0.1798	51
Chronicity index	0.003	0.9841	51
Creatinine	−0.078	0.5868	51

*All statistical *p*-values (*p* < 0.05) are bold.*

### C4d Levels Vary Between the Types of LN

**FIGURE 3:**
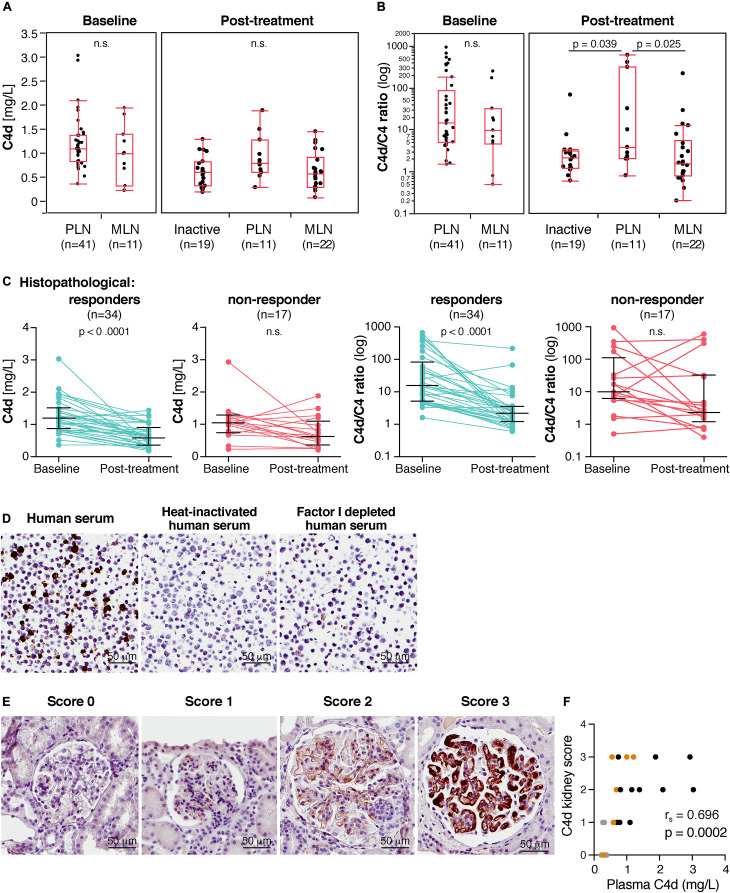
In lupus nephritis (LN) patients, C4d levels associate with histopathological responsiveness to treatment and plasma C4d levels correlate with C4d deposited in kidney biopsies. **(A)** C4d levels and **(B)** C4d/C4 ratio at baseline and post-treatment of LN subgroups. Inactive patients represent ISN class I, II and III C. **(C)** C4d levels and C4d/C4 ratio at baseline and post-treatment in histopathological responders and non-responders. Each patient sample plus the medians and interquartile ranges are shown. **(D)** Specificity of C4d staining with anti-C4d-neo monoclonal Ab was determined by immunohistochemistry of Daudi cell pellets incubated with OmCI, ofatumumab and 5% human serum. Heat-inactivated human serum (5%) and 5% factor I depleted sera were applied as negative controls not able to generate C4d. **(E)** Scoring of C4d levels in kidney biopsies of LN patients. Representative images for each score at 40× magnification are presented. **(F)** Correlation of C4d plasma levels with C4d deposition in kidney biopsies. PLN samples are shown in black, MLN samples in orange and inactive samples in gray. Significance was calculated using Kruskal-Wallis rank-sum and Mann–Whitney U tests **(A,B)**, Wilcoxon matched-pairs signed rank test **(C)** and Spearman’s rank-order correlation test **(F)**. PLN, proliferative LN; MLN, membranous LN.

In order to investigate C4d in regard to the responsiveness to various treatments, C4d levels were determined at baseline and post-treatment in 52 LN patients. At baseline, all patients had an active LN and according to biopsies, 41 cases were classified as proliferative LN (class III/IV ± V) and 11 cases as pure membranous LN (class V) (for detailed information see Table 1). At baseline, C4d levels did not differ significantly between the proliferative (1.1 mg/L, 0.36–3.03 mg/L) and membranous (1 mg/L, 0.23–1.95 mg/L, *p* = 0.244) LN patient subgroups (Figure 3A).

Post-treatment, a significant reduction in C4d levels was observed in the total group (*p* < 0.0001, Table 5) and repeated biopsies were classified into three groups. Inactive biopsy findings (*n* = 19) consisted of patients with either class I, II or III (C). There were 11 patients still showing a proliferative pattern (class III and IV+V) and 22 patients with membranous LN (class V). In three of these, a concomitant class II was seen. C4d levels in the proliferative LN patients (0.78 mg/L, 0.28–1.88 mg/L) were higher than in the membranous LN patients (0.57 mg/L, 0.07–1.44 mg/L, *p* = 0.064) and than in the inactive patients (0.59 mg/L, 0.18–1.28 mg/L, *p* = 0.085). However, the differences did not reach statistical significance (Figure 3A).

**TABLE 5:** Clinical, laboratory and histopathological characteristics at first and second biopsy of the 52 re-biopsied LN patients.

Characteristics	Baseline	Post-treatment	*P* Value
Age	31.5 (18–62)		
Females, n (%)	45 (87%)		
Ethnicity, n Caucasian African Asian Hispanic Middle East	40 1 5 3 3		
C4d (mg/L) LN all (*n* = 52, paired) Inactive (*n* = 0/19) Proliferative LN (*n* = 41/11) Membranous LN (*n* = 11/22)	1.1 (0.23–3.03) – 1.1 (0.36–3.03) 1 (0.23–1.95)	0.61 (0.07–1.88) 0.59 (0.18–1.28) 0.78 (0.28–1.88) 0.57 (0.07–1.44)	**<0.0001** – 0.059 0.123
C4d (mg/L) Histopathological responders (*n* = 35) Histopathological non-responders (*n* = 16)	1.15 (0.24–3.03) 1.04 (0.23–2.93)	0.59 (0.07–1.44) 0.66 (0.21–1.88)	**<0.0001** 0.071
C4d (mg/L) – Clinical response Complete responders (*n* = 25) Partial responders (*n* = 10) Non-responders (*n* = 17)	1.1 (0.33-3.03) 1.12 (0.67–1.82) 1.07 (0.23–2.93)	0.54 (0.23–1.44) 0.6 (0.18–1.09) 0.66 (0.07–1.88)	**<0.0001 0.002 0.045**
C3 (g/L)	0.54 (0.12–1.38) 5 missing	0.77 (0.34–1.31) 8 missing	**<0.0001**
C4 (mg/L)	85 (3.15–554)	281 (3.15–744)	**<0.0001**
C4d/C4 ratio LN all (*n* = 52, paired) Inactive (*n* = 0/19) Proliferative LN (*n* = 41/11) Membranous LN (*n* = 11/22)	14.5 (0.5–929) − 14.5 (1.5–929) 9.8 (0.5–251)	2.2 (0.2–597) 2.1 (0.6–67.3) 3.8 (0.8–597) 1.55 (0.2–217)	**<0.0001** − 0.219 **0.023**
Creatinine (μmol/L)	75 (32–173)	71 (33–159)	0.089
Urine-albumin to creatinine ratio (mg/mmol)	126 (0.8–556) 27 missing	5.6 (0–503) 26 missing	0.064
Anti-dsDNA Ab positive, n (%)	36 (84%) 9 missing	22 (73%) 17 missing	
Renal histology (ISN/RPS), n Inactive [class I, II or III (C)] Proliferative [class III/IV ± V] Membranous [class V]	0 41 11	19 11 22	
Activity index	5 (0–12)	1 (0–10)	**<0.0001**
Chronicity index	0 (0–6)	1 (0–9)	**0.017**
Prednisolone, n (%) (mg/day)	36 (69%) 7.5 (0–60)	51 (1%) 10 (0–40)	
Antimalarials, n (%)	17 (33%)		
DMARD at biopsy, n (%)	Any 14 (27%) AZA 7 MMF 4 CYC 1 MTX 2		
Treatment after first biopsy	MMF 17 CYC 19 MMF/CYC switched 4 RTX 7 RTX combined with other DMARD 4 AZA 1		

*All statistical significant *p*-values (*p* < 0.05) are bold. Values are presented as median (range) unless otherwise stated. LN, lupus nephritis; AZA, azathioprine; CYC, cyclophosphamide; DMARD, disease-modifying anti-rheumatic drugs; MF, mycophenolate mofetil; MTX, methotrexate; RTX, rituximab; n.d., not determined.*

The C4d levels of the 11 patients with remaining PLN findings post-treatment were not significantly lower than for the 41 patients with PLN at baseline (Table 5). The same was observed for the MLN patients.

At baseline, the median C4d/C4 ratio was also higher in proliferative (14.5, 1.5–929) than in membranous (9.8, 0.5–251) LN patients. However, likewise for C4d levels, the difference did not reach significance (*p* = 0.426) (Figure 3B). Post-treatment, the C4d/C4 ratio was significantly higher in proliferative LN than in inactive patients (*p* = 0.039) and than in membranous LN patients (*p* = 0.025), where differences in C4d levels alone did not reach significance.

The C4d/C4 ratio of the 11 patients with remaining PLN post-treatment was not significantly lower than for the 41 patients with PLN at baseline. However, the C4d/C4 ratio was significantly lower post-treatment in the MLN patients compared to baseline levels (Table 5).

### C4d Levels Associate With Histopathological Responsiveness

Following treatment, 34 patients (65%) met the criteria of histopathological response and 17 patients (33%) were defined as histopathological non-responders. One patient could not be classified due to insufficient quality of the biopsy. Strikingly, C4d levels as well as the C4d/C4 ratio (Figure 3C) decreased significantly (*p* < 0.0001) in the histopathological responders, but did not change in the non-responders (C4d, *p* = 0.0714; C4d/C4 ratio, *p* = 0.2842).

### Plasma C4d Levels Correlate With C4d Deposition in Kidneys

Specificity of anti-C4d-neo monoclonal Ab was verified by immunohistochemistry using paraffin-embedded Daudi cells incubated with OmCI, ofatumumab and human serum. Heat-inactivated human serum lacking active complement, and factor I depleted human serum, which cannot generate C4d from C4b, were used as negative controls (Figure 3D). C4d expression was investigated in matched renal biopsies at baseline and post-treatment of 12 LN patients. The staining intensity of C4d in the glomeruli was scored from zero to three (Figure 3E). The C4d staining pattern was segmental glomerular. Strikingly, plasma C4d levels correlated strongly with C4d deposited in LN kidneys (*r*_*s*_ = 0.696, *p* = 0.0002) (Figure 3F). The C4d/C4 ratio likewise correlates significantly with C4d deposited in LN kidneys (*r*_*s*_ = 0.637, *p* = 0.0011). Table 3 summarizes plasma C4d levels, kidney C4d scores as well as LN classes of the 12 re-biopsied patients.

### Plasma C4d Levels Associate With Clinical Responsiveness

**FIGURE 4:**
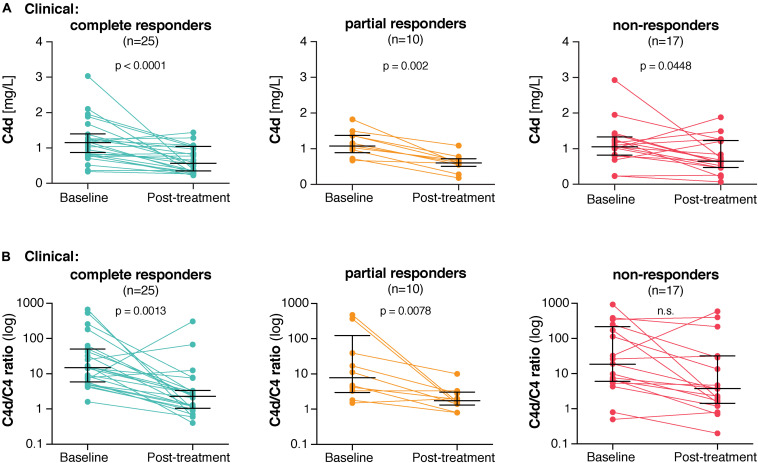
In lupus nephritis patients, C4d levels associate with clinical responsiveness to treatment. **(A)** C4d levels as well as **(B)** C4d/C4 ratios at baseline and post-treatment in clinical complete, partial and non-responders. Each patient sample plus the medians and interquartile ranges are shown. Significance was calculated using Wilcoxon matched-pairs signed rank test.

Twenty-five patients (48%) met the definition of complete clinical response, 10 patients (19%) of partial clinical response and 17 patients (33%) were categorized as clinical non-responders. C4d levels decreased most significantly in the clinical complete responders (*p* < 0.0001) and strongly in the clinical partial responders (*p* = 0.002), whereas the decrease in the clinical non-responders just reached significance (*p* = 0.045) (Figure 4A). Also, the C4d/C4 ratio associated most strongly with clinical complete responsiveness (*p* = 0.0013) and strongly with clinical partial responsiveness (*p* = 0.0078) (Figure 4B). However, no association with clinical non-responsiveness was observed (*p* = 0.051).

## Discussion

In the current study, we confirm our previous observation that plasma C4d is a valuable marker for LN (8). Furthermore, we revealed that plasma C4d levels correlate with the intensity of C4d deposition in kidney biopsies and that they associate with histopathological and clinical responsiveness to immunosuppressive treatment. Additionally, we show that the ratio of C4d over C4 might be even more valuable than either C4d or C4 alone as a marker for LN.

Despite significant advances in treatment options, LN is still one of the most severe manifestations in SLE and it accounts for an increased morbidity. LN patients have a worse prognosis and end stage renal failure is suggested to be an important cause of mortality in SLE (1). Various biomarkers have been suggested to monitor disease activity, to forecast general flares and/or exacerbations of specific manifestations including LN and to verify treatment response. However, no biomarker covers all of these aspects, which might be due to the extraordinary clinical complexity of SLE and the fact that each individual patient exhibits a distinct course of the disease (18). Neither albuminuria, levels of anti-DNA antibodies nor complement levels, which are commonly used for evaluation of renal response, proved to be reliable biomarkers in LN (7). Among biomarkers, serum levels of soluble Axl were recently proposed as useful marker to reflect the grade of inflammation and tissue damage in the kidneys as well as to assess histological response to immunosuppression in LN (19). Among urinary biomarkers, the activated leucocyte cell adhesion molecule (ALCAM) could distinguish between renal and non-renal SLE and high baseline urinary ALCAM levels seemed to increase the risk for deterioration of renal function (20).

The pathogenesis of LN involves immune complex deposition (21), which strongly activate complement and thus incite inflammatory mechanisms contributing to the severity of the disease. Importantly, low levels of C3 and C4 are included in the SLE disease activity index 2000 (SLEDAI-2K), which is commonly used to monitor SLE (14). However, C3 and C4 exhibit wide reference ranges in healthy individuals (22), have low sensitivity in follow-up of SLE patients and were unable to predict the recurrence of LN as stand-alone markers (23). In addition, low C3 and C4 levels do not accurately mirror complement activation, since their levels are also affected by the rate of synthesis, which further increases during inflammation. We have previously introduced a unique assay to quantify plasma levels of C4d, which is the final cleavage fragment of C4 exclusively arising from complement activation (9). This specificity is achieved by a neoepitope-specific antibody that is directed against a very short linear epitope in the cleavage site of C4d that becomes exposed solely after complement-mediated cleavage of C4b, and that cannot be mimicked by non-proteolytic events (9). By the use of this assay, we could confirm that C4d levels are increased in LN patients and that C4d is a valuable marker to discriminate LN from non-renal SLE. Previously, we showed that C4d levels were negligible in healthy individuals that were sampled under optimal conditions (8). In the current study, population-based controls were used for comparison and the C4d levels were as expected higher than in previously determined healthy controls, but still significantly lower than in SLE patients. Considering that these control individuals could have other diseases than SLE, and thus represent a more representative control cohort, the results show that C4d associates with SLE and especially with LN in relevant settings.

Previously, we discovered that C4d, but not C3 or C4, was an accurate marker to distinguish between remission and flare, as well as to predict future LN flares in relapsing patients in a cross-sectional SLE cohort (8). C4d levels were significantly higher in LN patients than in patients without renal involvement (8). This observation was confirmed in the current study. However, high C4d levels alone, even though exhibiting higher sensitivity, specificity, PPV, NPV and overall accuracy than low C4, were not statistically superior to low C4 levels, as it was the case in the previous study.

A clear strength of the current study is that all LN cases were biopsy verified and repeated biopsies allowed us to determine both histopathological and clinical outcome. Clinical responsiveness was defined using established outcome measures (2), although no general definition of histopathological outcome has been generally introduced. Applying similar histopathological response criteria from studies on repeat renal biopsies (7), we have previously identified high interferon lambda levels as a marker of poor treatment response (17) and now C4d levels associate even more strongly with treatment outcome.

According to the current treatment guidelines, LN patients should be treated with corticosteroids in combination with aggressive immunosuppressive drugs (4). Furthermore, angiotensin converting enzyme inhibitors (ACEi) or angiotensin receptor blockers (ARB) are generally recommended. Apart from being anti-hypertensive, ACEi/ARB reduce the amount of proteinuria, which in turn may influence the evaluation of renal response, proteinuria being one of the main clinical measures in the established response criteria (2). Thus, firm evaluation of response may be hampered by therapy itself, further stressing the need for a reliable biomarker that may reflect ongoing inflammation. At baseline biopsies, we found a clear correlation between C4d and the grade of albuminuria, most probably reflecting the renal activity. The lack of association at repeated biopsy could be explained by the use of ACEi/ARB, which lower the albuminuria to various degrees and thereby interfere with the evaluation of clinical response.

Here, we report that plasma C4d levels, as well as the C4d/C4 ratio, declined in LN patients with favorable clinical and histopathological response, but remained mostly unchanged in non-responders. This strongly suggests that C4d and the C4d/C4 ratio might be useful markers to study the responsiveness to immunosuppressive treatments. Although renal biopsies are the gold standard to diagnose and classify LN (24), follow-up biopsies after induction of immunosuppressive therapy are not and carry potential risks for bleeding complications (25). Since C4d levels as well as C4d/C4 ratios were shown to associate strongly with both clinical and histopathological responsiveness, our findings strongly suggest that the simple determination of plasma C4d might at least partially replace invasive biopsies. C4d levels correlated with the activity index at baseline but not post-treatment. Interestingly, despite effective immunosuppressive treatment, the chronicity index increased post-treatment. The findings indicate that C4d plasma levels may be a better measure for active LN than for chronic presentation and point to the fact that the inflammatory and thus scarring process persists despite intense immunosuppressive treatment.

The diverse manifestations and sample size in the non-renal SLE controls did not allow us to analyze associations between specific non-renal manifestations and C4d findings. However, despite having active disease, this patient group displayed clear difference in C4d compared to LN patients, further pointing to the role C4d in renal lupus.

C4 exists in two polymorphic forms, C4A and C4B, which exhibit distinct chemical reactivities. Partial C4 deficiency can arise due to copy number variations (C4 null alleles) as well as mutations (26). Notably, this does not only occur in SLE patients, but also in approximately 35% of the general population. However, deficiencies or low copy numbers of either total C4 or C4A are a risk factor for SLE development (27). In the case of partial C4 deficiency, low C4 levels are a consequence of decreased synthesis rather than increased complement activation. Accordingly, quantifying the C4d/C4 ratio might correct for this phenomenon. In a recent study of severe LN patients, that included patients on combination therapy of rituximab and belimumab, the C4d/C4 ratio correlated better with levels of anti-dsDNA and anti-C1q antibodies than stand-alone measurement of C4d or C4 (28). None of these markers correlated with the renal outcome criteria of proteinuria. However, the change in C4d/C4 ratio from baseline to post-treatment correlated to the change in proteinuria and outperformed sole quantification of C4d or C4. Importantly, no repeated biopsies were performed and thus no data on histopathological response are available.

The limitation of our study is the retrospective nature and that methods for detection of anti-dsDNA antibody levels were determined with various methods and missing data in a proportion of patients. Thus it is reasonable that we only detected a weak association of C4d levels with anti-dsDNA antibody occurrence in this study, whereas we previously found a strong association (8). Another limitation of the current study is that proteinuria was also quantified with different methods.

C4d is frequently used as a biomarker for antibody-mediated renal graft rejection (29). Besides the presence of donor-specific antibodies and histopathological evidence of tissue injury, C4d deposition in peritubular capillaries has become the cornerstone diagnostic parameter in graft rejection (30). In contrast to the peritubular deposition, we now found expected distinct glomerular C4d expression in LN patients. Although complement activation is an underlying pathogenetic mechanism in the two renal conditions, the localization of C4d differs and thus targets separate renal compartments.

Increasing numbers of complement inhibitors are in clinical trials and reliable assays for the determination of treatment response are a prerequisite for conclusive studies (31). Determination of the soluble terminal complement complex (sTCC) is a good measure for general complement activation, but not specific for the classical pathway. Other complement biomarkers, such as C3a and C5a are very short lived. Measurement of cell-bound C4d and C3d was previously suggested (32) but these assays are difficult to perform in clinical practice (flow cytometry using fresh patient blood). Thus, our cleavage neoepitope specific C4d antibody used in a fast and practical C4d assay might be a valuable tool not only in SLE, but also in other complement-mediated diseases.

By analyzing an independent SLE cohort, we here confirm previous findings regarding the role of C4d as a biomarker in LN. We demonstrate a correlation between C4d levels in plasma and C4d levels in kidney biopsies, as well as showing C4d as being a marker of treatment response, both at a clinical and histopathological level. If confirmed in larger studies, measurement of circulating C4d could contribute to assessment of LN activity and may lessen the need of renal biopsies in the future.

## Data Availability Statement

The raw data supporting the conclusions of this article will be made available by the authors, without undue reservation.

## Ethics Statement

The studies involving human participants were reviewed and approved by Regional Ethical Review Board in Stockholm. The patients/participants provided their written informed consent to participate in this study.

## Author Contributions

MM, RT, and SN performed the experiments and analyzed the results. IG and AZ acquired and analyzed clinical parameters. AB organized and diluted the samples. IG recruited patients and supervised the study together with MM and AMB, who also interpreted the data. MM drafted the manuscript together with AMB. All authors have read, revised and approved the final manuscript.

## Conflict of Interest

AMB is named as inventor in a patent application including claims to use of C4d as biomarker. The remaining authors declare that the research was conducted in the absence of any commercial or financial relationships that could be construed as a potential conflict of interest.
